# From the Wild to the Cup: Tracking Footprints of the Tea Species in Time and Space

**DOI:** 10.3389/fnut.2021.706770

**Published:** 2021-08-06

**Authors:** Moses C. Wambulwa, Muditha K. Meegahakumbura, Samson Kamunya, Francis N. Wachira

**Affiliations:** ^1^Department of Life Sciences, South Eastern Kenya University, Kitui, Kenya; ^2^Key Laboratory for Plant Diversity and Biogeography of East Asia, Kunming Institute of Botany, Chinese Academy of Sciences, Kunming, China; ^3^Genetics and Plant Breeding Division, Coconut Research Institute of Sri Lanka, Lunuwila, Sri Lanka; ^4^Kenya Agricultural and Livestock Research Organization, Tea Research Institute (KALRO-TRI), Kericho, Kenya

**Keywords:** tea, *Camellia sinensis*, footprints of tea, wild tea, origin of tea, tea domestication, expansion history

## Abstract

Tea is one of the world's most popular beverages, known for its cultural significance and numerous health benefits. A clear understanding of the origin and history of domestication of the tea species is a fundamental pre-requisite for effective germplasm conservation and improvement. Though there is a general consensus about the center of origin of the tea plant, the evolutionary origin and expansion history of the species remain shrouded in controversy, with studies often reporting conflicting findings. This mini review provides a concise summary of the current state of knowledge regarding the origin, domestication, and dissemination of the species around the world. We note that tea was domesticated around 3000 B.C. either from non-tea wild relatives (probably *Camellia grandibracteata* and/or *C. leptophylla*) or intra-specifically from the wild *Camellia sinensis* var. *assamica* trees, and that the genetic origins of the various tea varieties may need further inquiry. Moreover, we found that lineage divergence within the tea family was apparently largely driven by a combination of orogenic, climatic, and human-related forces, a fact that could have important implications for conservation of the contemporary tea germplasm. Finally, we demonstrate the robustness of an integrative approach involving linguistics, historical records, and genetics to identify the center of origin of the tea species, and to infer its history of expansion. Throughout the review, we identify areas of debate, and highlight potential research gaps, which lay a foundation for future explorations of the topic.

## Introduction

Tea is thought to be the world's most popular non-alcoholic beverage, with more than 2 billion cups consumed daily (1). The popularity of the tea beverage is mainly ascribed to its high nutritive value and health benefits. For instance, tea contains high amounts of mineral elements, sugars, amino acids, organic acids, and flavonoids (2–4), which impart various physiological benefits to consumers. Moreover, extracts from the tea plant are known to possess antimicrobial, anti-inflammatory, anti-cancer, and anti-diabetic properties (5–11), which are conferred by its polyphenolic secondary metabolites, particularly the catechins. These dietary and health benefits have increased the crop's global demand; tea is now cultivated in at least 50 countries worldwide, where it contributes immensely to the local economies. The total global production volume of tea in 2019 was 6,497,443 metric tons, with the five largest producers being China (~42.9% of the total production), India (~21.4%), Kenya (~7.1%), Sri Lanka (~4.6%), and Vietnam (~4.1%) (12).

The sociocultural and religious significance of tea has also contributed to its popularity. Within the Buddhist monasteries in Tibet, the offering of tea-ceremonies was a requirement for new monks, as well as a mark for certain key milestones for the serving monks (13). The famous Tea-Horse road (“Chamadao”), winding through southwest China (Sichuan, Yunnan, and Tibet), was an important route for trading tea and other products, and is today one of China's major sites of cultural heritage (14). Moreover, the Chinese tea ceremony is today rooted in the traditional wedding culture. In Assam, India, tea drinking is deeply entrenched in the life and culture of the local communities, particularly for religious and social events (15). In Japan, the tea ceremony is a very important and elaborate ritual that has a lot of meaning within the culture, with the tea beverage representing purity, tranquility, respect and harmony. Correspondingly, the introduction of tea to Britain in the seventeenth century caused a social revolution, replacing milk and ale as the most popular drinks then, and inculcating a new social etiquette across the country (16). This blending of dietary, medicinal, economic and sociocultural significance of tea has further enhanced its importance, both real and perceived, around the world.

The tea plant [*Camellia sinensis* (L.) O. Kuntze] is a perennial shrub of the Section *Thea* and Genus *Camellia* in the family Theaceace. Although species delimitation for *Camellia* remains generally unresolved, cultivated tea is mostly classified according to Barua (17), who recognized two cultivated species: *C*. *sinensis* (hereafter referred to as CSS), and *C*. *assamica* (Masters) (hereafter referred to as CSA). A subspecific rank *C*. *assamica* subsp. *lasiocalyx* (Planch ex Watt.) (hereafter referred to as CAL) was also defined. The origin and expansion of tea around the world have remained understudied, and therefore are still poorly understood. For instance, most literature sources generally point to an area in Southeast Asia as the center of origin of the tea plant, without considering the possibility of multiple centers of origin corresponding to the various tea varieties, as suggested by genetic data (18). This knowledge gap about the center of origin may subsequently retard the quest to identify the true wild progenitor(s) of tea, and also hinder attempts to map out the possible post-domestication expansion routes of the species. The search for the non-tea ancestor of the tea species is also a question for which no unequivocal answer is available. This obscurity may impede effective genetic conservation and improvement efforts in many tea-producing countries, particularly since the center of origin of a crop species often harbors the wild relatives, which are invaluable genetic resources for enriching modern cultivars (19). For a crop species with a wide biochemical spectrum like tea, such wild genetic resources could unlock tremendous potential for enhancing the nutritional and therapeutic profile of tea accessions around the world. Here, we provide a concise account of the current state of knowledge on the origin (geographical and genetic) and the spatio-temporal expansion of the tea species. We concurrently highlight areas of disagreement, identify key axes of debate among published studies, and suggest ways of resolving the outstanding questions, notably by the adoption of integrative approaches.

## Geographical Origin of Tea

**Figure 1:**
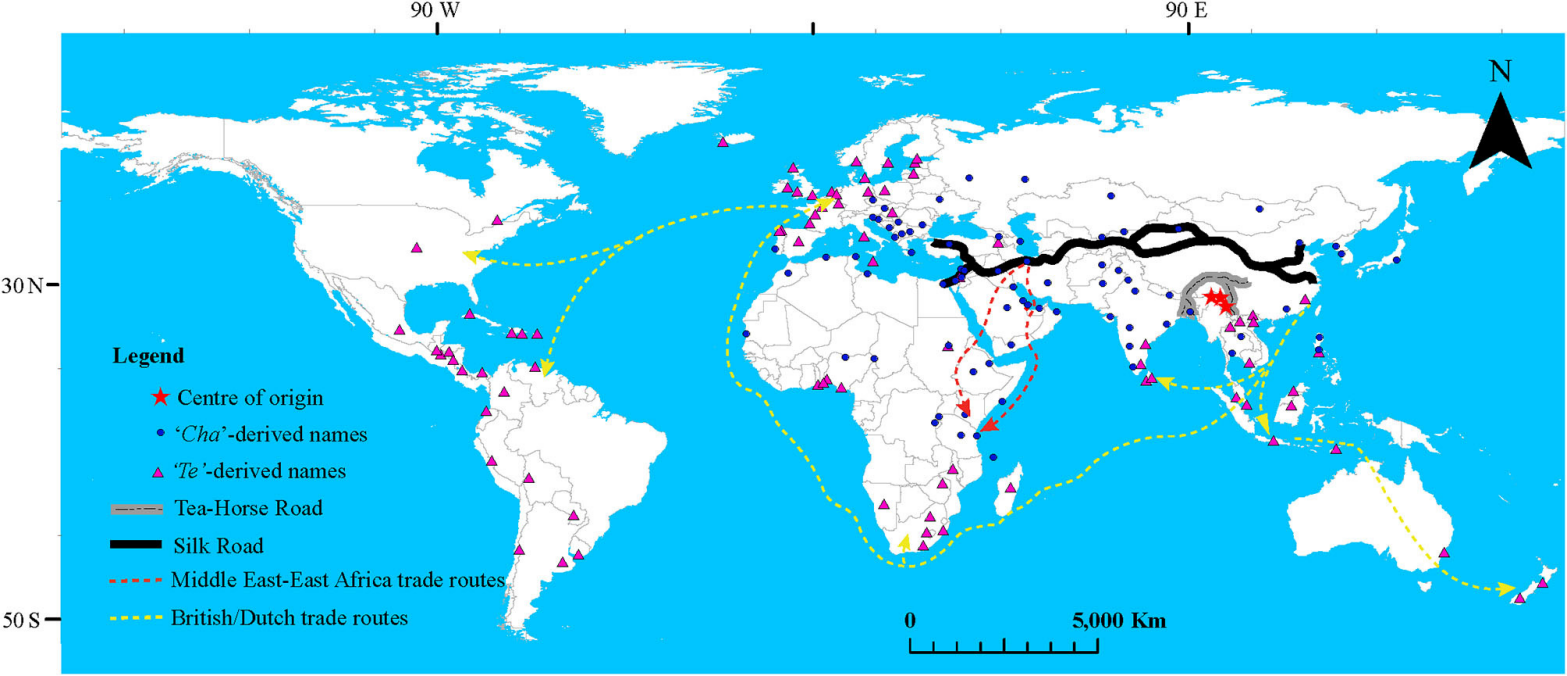
Linguistic and historical inference of the expansion routes of the tea plant and tea-drinking culture around the world beginning from the center of origin (Assam-Myanmar-Yunnan area). For convenience, mapping of the two phonetic forms was based on GPS locations of capital cities of countries where a given language is spoken, except for cases with multiple languages within a country. The map was generated using ArcGIS Desktop v10.7 (https://www.esri.com). KMZ files for the Silk and Tea Horse Roads were prepared in Google Earth Pro (https://www.google.com/earth), then converted to layer files in ArcGIS Desktop v10.7. The British/Dutch and Middle East-East Africa trade routes were approximated based on historical records.

The earliest literature sources on the origin of tea focus more on the drink, and much less on the plant, but generally point to China as the most probable origin. It is thought that tea drinking in China begun earlier than 2000 B.C., but its use as a popular commercial beverage would only gain momentum toward the end of the sixth century (20). Indeed, Chinese legends and ancient writings about tea do corroborate this view; for example, a legend from 2,737 B.C. credits the origin of tea drinking to Shen Nung, a Chinese emperor. Moreover, the earliest literary reference to tea, an ancient Chinese dictionary (*Erh Ya*), is dated 350 B.C., and was followed by the first monograph on tea in 780 AD [later translated to English as *The Classic of Tea*; (21)]. Subsequent works supported these earlier sources; for instance, Ward (22) speculated that the source of the Irrawaddy river might have served as the primary center of origin for the tea species. The various tea types were subsequently dispersed southwards to the Assam-Myanmar-Yunnan area (CSA and CAL), and southeastwards to eastern China (CSS) (22). The Assam-Myanmar-Yunnan area was thought to be the secondary center, from which there were further dispersal events in the general southern direction. Based on geological and biogeographical evidence, Yu (23) demonstrated that the tea plant originated from a narrow region between Wenshan and Honghe in Yunnan (between 22° 40′-24° 10′N and 103° 10′-105° 20′E). Later, Muramatsu (24) proposed that Wenshan and Xishuangbanna (both in Yunnan Province, China) are, respectively, the primary and secondary centers of tea origin. Although these previous accounts are equivocal about the origin of the tea plant, it appears that the area covering Southwest China, Northern Myanmar, and Northwest India is the putative center of origin of the tea species (Figure 1).

The early speculations by Ward (22) about the origin of CSS and CSA were later partly supported by some empirical genetic/genomic studies (18, 25, 26), which demonstrated that cultivated CSS and CSA might have different geographical origins. An emerging idea, however, is that CSA in China and India are genetically distinct (though morphologically similar), indicating independent geographic and evolutionary origin for the two CSA groups (18, 26). Although genetic data could be a powerful tool for inferring the geographic origin of a domesticated species, they only distinguish individuals based on their genetic similarity rather than their geographical origin [e.g., (27)], hence presenting a hurdle for meaningful inferences on geographic origin. Moreover, in cases where coding/non-neutral markers are used in the genetic analyses, long-distance dispersal followed by allopatric lineage divergence (as opposed to independent geographic origin) could be the underlying mechanism that explains the observed genetic variation in tea, especially in such a heterogenous landscape as Southwest China [e.g., (28, 29)]. Thus, future genetic explorations on the geographic origin of tea should apply high density neutral markers [e.g., genomic single nucleotide polymorphisms (SNPs)] derived from widely sampled populations in both Assam and Southwest China; such markers would shed light on the fine details of the genetic relationships, hence might help to clarify some aspects regarding geographic origins. In addition, useful insights on the geographic origin of tea can be obtained through ecological niche modeling (ENM), a probabilistic approach that relies on environmental data to predict the spatio-temporal distribution of a given species [e.g., (30)]. However, for the tea species, conclusions from any ENM analysis must be interpreted with caution as the species has undergone extensive and intensive post-domestication artificial selection and breeding, and may therefore violate the “niche conservatism” assumption.

## Domestication History of Tea

The existence of wild tea trees [*C*. *sinensis* (L.) O. Kuntze] in China and India could mean that tea was domesticated directly from these wild tea trees, as opposed to the popular view that non-tea wild species were involved [e.g., (26), and references therein]. However, the plausibility of involvement of a non-tea ancestor seems to be validated by the fact that some non-tea *Camellia* species such as *C*. *crassicolumna* Chang, *C*. *gymnogyna* Chang, *C*. *tachangensis* F.C. Zhang, and *C*. *taliensis* (W.W. Smith) Melchior, are still consumed locally as “tea” in some parts of Southwest China, particularly in Yunnan Province. As interspecific hybridization within *Camellia* is possible (31, 32), and since the morphological features of the arising hybrids are often continuous, it is fairly difficult to trace modern tea to any single wild progenitor from which it might have been domesticated. This challenge notwithstanding, considerable research efforts have been made toward identifying the wild ancestor(s) of cultivated tea.

One of the key questions relating to tea domestication is whether the two tea types (CSS and CSA) evolved independently, or whether one was selected from the other. If they evolved independently, did they descend from the same, or two different ancestors? Ward (22) suggested that the three tea types (CSS, CSA, and CAL) might represent three independent domestication events, a view that is consistent with the findings of Chen and Yamaguchi (33), which placed CSS and CSA in separate genetic clusters. Wood and Barua (34) later demonstrated, based on chromatograms of phenolic constituents, that CSS might have descended from *C*. *irrawadiensis*. Relatively recent work suggested that *C*. *taliensis* could have been involved in the domestication of CSA in Yunnan (35), though the study might have been limited by the non-inclusion of all tea types, insufficient number of microsatellite markers, and the narrow geographic coverage. Huang et al. (36) and Meegahakumbura et al. (37) overcame some of these limitations by sampling the CSS and CSA tea types, as well as wild non-tea *Camellia* species covering most of the genus's phylogenetic diversity. Bayesian clustering and phylogenetic analyses in these studies (36, 37) indicated that the Chinese CSA tea may have been domesticated from *C*. *grandibracteata*, and CSS from *C*. *ptilophylla* or *C*. *leptophylla*. These findings were generally consistent with results of plastomic analyses of species in *Camellia*/Theaceae (26, 38–40), further reinforcing the view that *C*. *grandibracteata* and *C*. *leptophylla* might have been involved in the domestication of tea. Although the above studies did shed some light on the evolutionary origin of tea, controversies still abound; for instance, the phylogeny of Huang et al. (36) indicated that CSS and CSA might have undergone independent domestication from two different progenitors, while Rawal et al. (40) suggested that CSS may have descended from CSA. Therefore, future phylogenetic analyses should incorporate the entire phylogenetic spectrum of the Genus *Camellia*, as well as sufficient samples of both CSS and CSA from their entire distribution ranges in order to determine their evolutionary histories. Moreover, although some studies suggested that CAL could have a hybrid origin (18, 41), it is still unclear whether it originated from the hybridization between CSS and CSA or between Indian and Chinese CSA. Nuclear genomic data from across the three tea types could provide clearer insights into the evolutionary origin of CAL.

Despite the controversy around the subject of tea domestication, historical record shows that tea cultivation started in Sichuan Province, China, long before any other region in the world adopted the practice, probably about 3000 B.C. (22, 32). Consistently, tea plant remains from Chang'an (Xi'an, China) indicated that cultivation of tea begun at least 2100 years ago (42). Although the question of the precise timing of the onset of tea cultivation has received little research attention, and therefore remains poorly understood, the evolutionary divergence times among the various cultivated tea groups could serve as proxies for estimating the time when tea cultivation begun. For instance, genetic data show that Chinese CSA and Indian CSA might have diverged ~2.7 Kya (thousand years ago) (25), a time that corresponds well with the ancient legends and anecdotal evidence about the onset of tea drinking in China. A combination of these lines of evidence therefore indicates that shortly after tea domestication, mechanisms such as migration and geographic isolation might have resulted in lineage divergence within the cultivated germplasm, hence the emergence of the various tea types that exist today.

**Figure 2:**
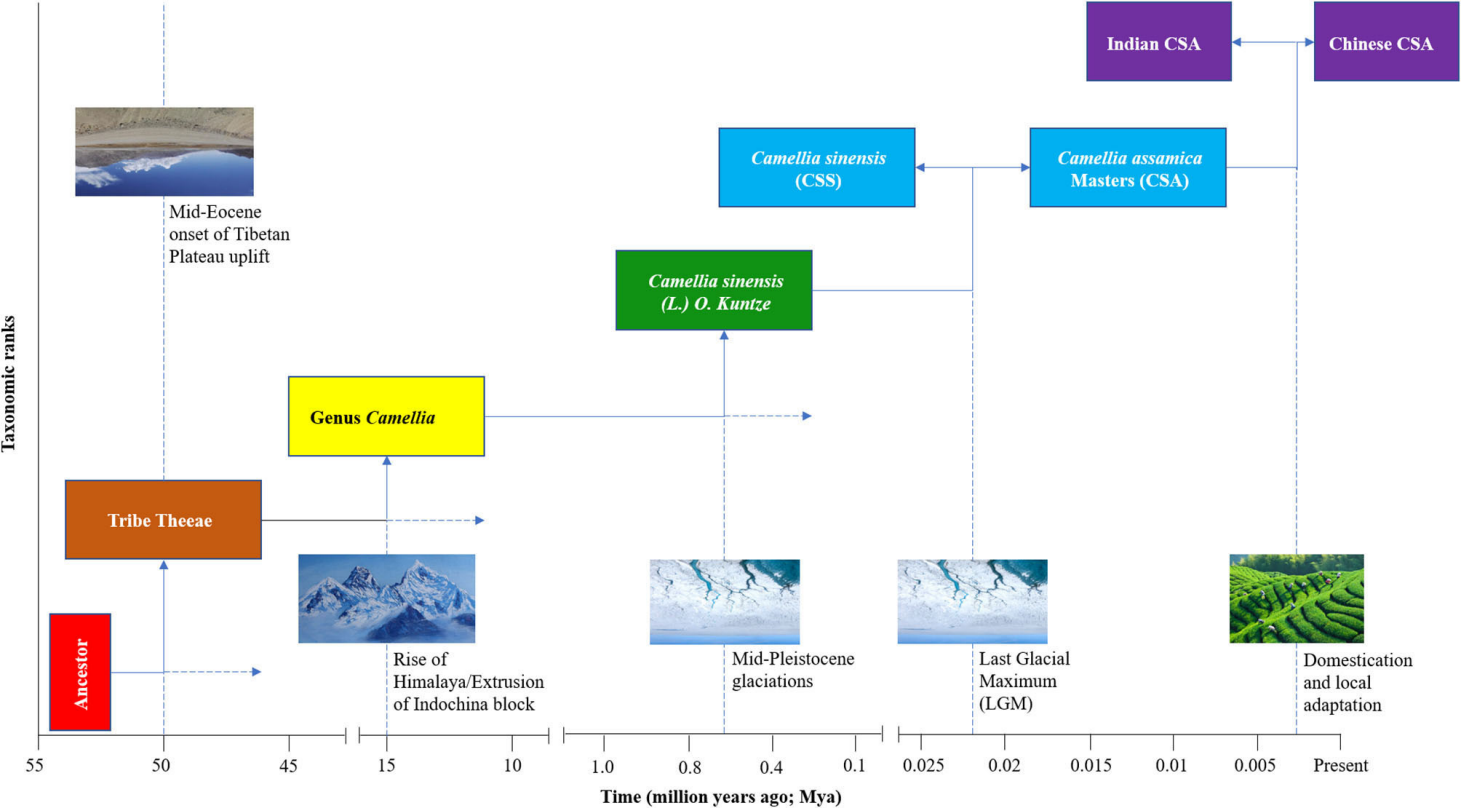
A schematic illustration of the evolutionary history of cultivated tea since the divergence of Tribe Theeae. The putative drivers of the divergence events are shown on the dotted lines extending from the time point of the divergence. The divergence events for Tribe Theeae, Genus *Camellia*, and Species *Camellia sinensis* (L.) O. Kuntze are based on a dated phylogeny by Yu et al. (38), while the subsequent lineage divergence within cultivated tea are based on a Bayesian computation modeling analysis by Meegahakumbura et al. (25). The images were obtained from Google Images: Himalaya (https://www.amazon.com/Gorkha-Everest-Himalayas-Original-Painting/dp/B07G9F6HC7), Glacier: (https://www.sciencemag.org/news/2020/07/greenland-drilling-campaign-aims-bedrock-trace-ice-sheet-s-last-disappearance), Cultivation (https://gracetea.com/an-introduction-to-tea).

To fully understand the evolutionary forces that gave rise to the tea plant as we know it today, it is important to first consider the pre-domestication natural selective forces that were in action during the evolutionary history of the tea plant. Dated phylogenies of Theaceae indicate that Tribe Theeae, Genus *Camellia* and Species [*Camellia sinensis* (L.) O. Kuntze] diverged from their ancestors ~50 Mya (million years ago), ~15 Mya, and ~625 Kya, respectively (38). The two time periods generally coincide with onset of the Tibetan Plateau uplift, rise of the Himalaya/extrusion of Indochina block, and the Mid-Pleistocene glaciations [(43–45); Figure 2], events that are thought to have influenced the distribution of plant species and driven lineage divergence in the region (46–49). Furthermore, genomic analyses and Bayesian computation models indicate that CSS and CSA diverged from their last common ancestor in the middle to upper Pleistocene (25, 50). Indeed, Pleistocene glaciations, which peaked ~21 Kya (Last Glacial Maximum, LGM; Figure 2), are known to have driven lineage divergence in many plant taxa across the Northern hemisphere, including Southwest China (51–53). The subsequent divergence of Indian CSA from Chinese CSA was constrained to ~2.7 Kya, a timeline that is generally consistent with legends and historical estimations on the onset of tea use and cultivation in China (25). These pieces of evidence indicate that orogenic, climatic, and anthropogenic factors might have jointly contributed to the evolution of the tea plant, and could partly explain agro-ecological preference of the tea plant for relatively cold and high-altitude areas.

Following its domestication, the tea plant underwent intensive cycles of further breeding and selection in order to improve its capacity to withstand biotic and abiotic stresses, as well as to enhance its organoleptic attributes such as aroma, taste, color, and texture (32, 54). For instance, recent transcriptomic and genomic analyses suggest that metabolic pathways in tea plants (particularly flavonoid biosynthetic pathways) underwent strong selection during the domestication process (50, 55, 56). Similarly, flavor and stress (biotic and abiotic) tolerance are among the main traits that were selected for during tea domestication or improvement processes (57). Wang et al. (57) found significant selective sweeps in CSS tea accessions for cytochrome P450 genes and terpene synthases, both of which are involved in flavor metabolism. Moreover, strong selection was also evident in CSS accessions for NBS-ARC (nucleotide-binding site domain in apoptotic protease-activating factor-1, R proteins and *Caenorhabditis elegans* death-4 protein) genes, which are associated with resistance to certain bacterial pathogens, and were upregulated during spring, autumn, or winter. These findings rekindle the debate on the evolutionary origin of the two main tea types (CSS and CSA). It seems plausible that CSS (adapted to the relatively colder higher latitudes) was selected from the wild CSA (adapted to a sub-tropical climate) in order to enhance CSS's survival in the harsher northern temperate climate. One of the key traits that was likely selected in CSS is the small leaf size, which is thought to confer a selective advantage to plants at higher latitudes (58). We argue that as CSA spread northwards within China, and later to Korea and Japan, cold tolerance became a necessary trait for survival, thus persistent selection for this attribute eventually produced CSS. This idea is reinforced by the fact that CSA has a relatively higher catechin content (56, 59), a phenotype that was recently associated with a primitive genotype predominantly found at the center of tea origin in Yunnan (55). Further insights on the precise genetic mechanism underlying tea domestication can be obtained using genome-wide association studies (GWAS) on a world-wide collection of tea accessions alongside *Camellia* wild species, as recently applied in clarifying the domestication history of castor bean (60).

## Expansion History of Tea Around the World

It might be difficult to distinguish between the spread of the tea plant and that of the tea products/drinking culture. For this reason, our discussion on this subject will be generalized to collectively encompass the biological (artificial) dispersal, trading of tea products, as well as introduction of the tea-drinking culture around the world. The spread of tea within the present-day China was mainly facilitated by the Yunnan-Tibet Tea Horse Road, which was established in the Sixth century [(61); Figure 1]. Subsequently, spread from China to the rest of the world began in the early Eighth century. Within Asia, tea was first introduced to Japan and onwards to Indonesia (32), with China being the likely ultimate source. Tea was then introduced to Europe in 1768 (62, 63) and later to Sri Lanka in 1839, from India in both cases. From Europe, the crop spread to Africa at the end of the Nineteenth century, though certain anonymous records indicate an earlier date of introduction (64). These early destination points subsequently served as dissemination centers to other parts of the world. To buttress these historical records, several studies have attempted to track the dissemination routes of tea around the world. For instance, the discovery of tea plant fossils along the Silk Road at Chang'an, China, supported the view that tea was introduced to the Middle East from China around the Second century (42). Additionally, useful insights have been gained from genetic data regarding the migration routes of the tea plant to Africa, with India being the main source (65). However, the outbreeding nature of tea means that most of the presently cultivated tea accessions around the world are hybrids, with varying genetic admixture proportions of the CSS, CSA. Since the interbreeding events were spread wide in space and time, genetic analysis alone cannot clarify the migration patterns and their associated timelines.

Owing to its popularity, long history of usage, and diverse roles (cultural, religious, economic, and therapeutic), the origins and distribution history of tea can only be fully understood through a multidisciplinary approach, as advised early on by Ward (22). Such an approach may incorporate disciplines such as plant biochemistry, genetics/genomics, paleontology, history, linguistics, as well as social anthropology. Linguistics, for instance, could be useful in understanding the expansion history of the tea crop and the tea drinking culture. This is possible because there are only two main phonetic forms of the names that refer to tea across the world (“*Cha*” -derived and “*Te*” -derived forms; https://translate.google.cn/?hl=en; accessed April 13, 2021), with only a few exceptions (Supplementary Table 1). The Cantonese “*Cha*” form, which was/is the most popular name for tea in China, might have spread along the Silk Road to Persia, and onwards to East Africa (possibly by Arab traders), and Eastern Europe (Figure 1). It is also likely that the “*Cha*” form spread along the Tea Horse Road to India via Tibet, and along the southern Silk Road to Laos and Thailand. On the other hand, the Min Nan (Fukian) “*Te*” form, then spoken in Fujian, China, was likely spread by the seafaring Europeans through the Dutch and British trading companies to Southeast Asia, Sri Lanka, Southern Africa, and Eastern Europe (Figure 1). The British East India Company could also be responsible for the introduction of the “*Cha*” form to East Africa from India, as the company's trade route was often along the East African coast (https://www.britannica.com/topic/East-India-Company; accessed April 16 2021).

These two nominal variants referring to tea can be used to infer the routes of expansion of the tea species, products, and drinking culture, thus augment the available evidence from historical records, fossil data, and genetics (41, 42, 65–69). For instance, some of these studies indicate that the tea grown in East Africa is predominantly Indian CSA, while that in Southeast Asia CSS, suggesting that the two tea types were introduced via the Silk Road and the European voyages in the Indian ocean, respectively. However, it appears that the Chinese CSA is still restricted to Southwest China and is yet to be utilized in breeding programs outside China. Although such an integrated approach is a powerful tool to deduce the historical dissemination of the tea plant, deeper insights could be gained from a genomic and morphometric analysis of a worldwide collection of tea accessions alongside wild tea trees whose intraspecific taxonomic status is known.

## Conclusion

We found that literature on the origin and early dissemination patterns of tea comprises a mix of legends, anecdotes, and empirical accounts, hence it is reasonably hard to delineate myth from fact. Nonetheless, the current review has shed light on the available scientific evidence, thus clearing away some of the misconceptions that have persisted for decades on the subject. Although our review has clarified the temporal aspects of tea origin and domestication, some questions about the spatial and evolutionary origin remain unanswered, particularly when considering the subspecific ranks of the species. Understanding the spatial and genetic origin of the tea species in this manner is restricted by the interfertility within the *Camellia* genus, as well as the outbreeding nature of the tea plant. The interfertility within *Camellia* has resulted in a proliferation of interspecific and intervarietal hybrids that are invariably morphologically similar to one of the parents. These shortcomings notwithstanding, the current review provides a brief spatio-temporal account of the domestication and dissemination of the tea species. It also crystalizes the key outstanding questions on the subject, and proposes specific hypotheses that can be tested directly in future studies. In addition to the aforementioned research gaps, future research on tea should explore the potential effect of climate change (particularly temperature and rainfall patterns) on the tea biochemical profile, as tea plants are thought to be highly sensitive to environmental variations (70, 71). Secondly, we propose the upscaling of the utilization of the Chinese CSA in global breeding programs, especially through international germplasm exchange initiatives. These additional interventions will supplement the existing research efforts and collectively contribute toward nurturing a resilient and sustainable tea enterprise, whose germplasm also caters for the nutritional needs of the billions of tea drinkers around the world.

## Author Contributions

MW and MM conceptualized the manuscript. MW undertook the literature review and wrote the manuscript. MM, SK, and FW reviewed and edited the manuscript. All authors contributed to the article and approved the submitted version.

## Conflict of Interest

The authors declare that the research was conducted in the absence of any commercial or financial relationships that could be construed as a potential conflict of interest.

## Publisher's Note

All claims expressed in this article are solely those of the authors and do not necessarily represent those of their affiliated organizations, or those of the publisher, the editors and the reviewers. Any product that may be evaluated in this article, or claim that may be made by its manufacturer, is not guaranteed or endorsed by the publisher.
