# Supplementary material for: From the Wild to the Cup: Tracking Footprints of the Tea Species in Time and Space
Source: Front Nutr. 2021 Aug 6;8:706770. doi: 10.3389/fnut.2021.706770 (PMC8377202; doi:10.3389/fnut.2021.706770)
Supplement: Supplementary file 1 [file Table_1.DOCX]

**Table S1:** Name for tea in 98 languages across the world. The language translations were accessed from Google Translate (https://translate.google.cn/?hl=en). Serial numbers 1-50 show the Fukian '*Te*' derivatives (first syllable pronounced with 't' sound), while serial numbers 51-98 show the Cantonese '*Cha*' derivatives (first syllable pronounced with 'ch', 'sh', or 's' sounds).

| **S/No.** | **Language** | **Name for 'tea'** |
| --- | --- | --- |
| 1 | Afrikaans | Tee |
| 2 | Armenian | T’ey |
| 3 | Basque | Tea |
| 4 | Chichewa | Tiyi |
| 5 | Chinese (fujian) | Te |
| 6 | Corsican | Tè |
| 7 | Danish | Te |
| 8 | Dutch | Thee |
| 9 | English | Tea |
| 10 | Esperanto | Teo |
| 11 | Estonian | Tee |
| 12 | Finnish | Teetä |
| 13 | French | Thé |
| 14 | Frisian | Tee |
| 15 | Galician | Té |
| 16 | German | Tee |
| 17 | Haitian Creole | Te |
| 18 | Hawaiian | Tī |
| 19 | Hebrew | The |
| 20 | Hmong | Tshuaj Yej |
| 21 | Hungarian | Tea |
| 22 | Icelandic | Te |
| 23 | Igbo | Tii |
| 24 | Indonesian | Teh |
| 25 | Irish | Tae |
| 26 | Italian | Tè |
| 27 | Javanese | Teh |
| 28 | Khmer | Te |
| 29 | Latvian | Tēja |
| 30 | Luxembourgish | Téi |
| 31 | Malagasy | Dite |
| 32 | Malay | Teh |
| 33 | Maltese | Te |
| 34 | Maori | Tī |
| 35 | Norwegian | Te |
| 36 | Samoan | Ti |
| 37 | Scots Gaelic | Tì |
| 38 | Sesotho | Tee |
| 39 | Shona | Tii |
| 40 | Sinhala | Tē |
| 41 | Spanish | Té |
| 42 | Sundanese | Téh |
| 43 | Swedish | Te |
| 44 | Tamil | Tēnīr |
| 45 | Telugu | Tēnīru |
| 46 | Vietnamese | Trà |
| 47 | Welsh | Te |
| 48 | Xhosa | Iti |
| 49 | Yoruba | Tii |
| 50 | Zulu | Itiye |
| 51 | Albanian | Çaj |
| 52 | Amharic | Shayi |
| 53 | Arabic | Shay |
| 54 | Azerbaijani | Çay |
| 55 | Bengali | Cā |
| 56 | Bosnian | Čaj |
| 57 | Bulgarian | Chaĭ |
| 58 | Cebuano | Tsaa (Cha) |
| 59 | Chinese (cantonese) | Chá |
| 60 | Croatian | Čaj |
| 61 | Czech | Čaj |
| 62 | Filipino | Tsaa (Chaa) |
| 63 | Georgian | Chai |
| 64 | Greek | Tsái |
| 65 | Gujarati | Cā |
| 66 | Hausa | Shayi |
| 67 | Hindi | Chaay |
| 68 | Japanese | Ocha |
| 69 | Kannada | Cahā |
| 70 | Kazakh | Şay |
| 71 | Kinyarwanda | Icyayi |
| 72 | Korean | Cha |
| 73 | Kyrgyz | Çay |
| 74 | Lao | Sa |
| 75 | Macedonian | Čaj |
| 76 | Malayalam | Cāya |
| 77 | Marathi | Cahā |
| 78 | Mongolian | Tsai (Sai) |
| 79 | Nepali | Ciyā |
| 80 | Odia (Oriya) | Cha |
| 81 | Pashto | Chai |
| 82 | Persian | Chai |
| 83 | Portuguese | Chá |
| 84 | Punjabi | Cāha |
| 85 | Romanian | Ceai |
| 86 | Russian | Chay |
| 87 | Serbian | Čaj |
| 88 | Slovak | Čaj |
| 89 | Slovenian | Čaj |
| 90 | Somali | Shaah |
| 91 | Swahili | Chai |
| 92 | Tatar | Shey |
| 93 | Thai | Chā |
| 94 | Turkish | Çay |
| 95 | Ukrainian | Chay |
| 96 | Urdu | Chai |
| 97 | Uyghur | Chai |
| 98 | Uzbek | Choy |
